# Impact of the reporting source on Platelet Inhibition and Treatment Outcomes (PLATO) trial deaths

**DOI:** 10.15190/d.2023.13

**Published:** 2023-09-25

**Authors:** Victor Serebruany, Jean-Francois Tanguay, Hector A. Cabrera-Fuentes, Milana L. Gurvich, Thomas Marciniak

**Affiliations:** Johns Hopkins University, Department of Neurology, Baltimore, MD, USA; Montreal Heart Institute, Université de Montréal, Montreal, Quebec, Canada; Research Center, Faculty of Medicine UNAM-UABJO, Autonomous University “Benito Juárez” of Oaxaca (UABJO), Oaxaca, Mexico; University of Maryland, College Park, Maryland USA; Bethany Beach, Delaware, USA

**Keywords:** Clinical trial, ticagrelor, clopidogrel, death, mortality.

## Abstract

BACKGROUND: Platelet Inhibition and Clinical Outcomes (PLATO) was a multicenter, randomized double-blind trial assessing efficacy and safety of ticagrelor versus clopidogrel in patients with acute coronary syndrome. The reported mortality benefit of ticagrelor in the PLATO trial has been challenged for over decade, and never confirmed in later trials.OBJECTIVE: To compare if there were any differences when deaths were reported to the FDAby the sponsors or by independent Contract Research Organizations (CRO).METHODS: We obtained the complete PLATO deaths dataset reported to the FDA and revealed that some events were inaccurately reported favoring ticagrelor. The entire FDA list contains precisely detailed 938 PLATO deaths. The CRO reported outcomes from the USA, Russia, Georgia, and most of Ukraine, while sites in 39 other countries were controlled by the trial sponsors. We compared vascular- (code “11”), non-vascular- (code “12”), and unknown (code “97”) deaths triaged by the reporting source.RESULTS: Overall, most PLATO deaths were vascular (n=677), less non-vascular (n=159) andunexpectedly many of “other” (n=7) or “unknown” (n=95) origin reported either by sponsors (n=807) or CRO (n=131). The trial sponsors reported more clopidogrel deaths from vascular (313 vs.239), non-vascular (86 vs.58) and unknown (53 vs. 26) causes.In contrast, CRO-monitored sites reported significantly (72 vs. 53; p<0.01) more ticagrelordeaths than after clopidogrel from vascular (51 vs.39), non-vascular (8 vs.7) and unknown (10 vs. 4) causes.CONCLUSION: Deaths were reported differently by sponsors and CRO within the same trial. Since some deaths were misreported by PLATO sponsors, only the CRO data seems mostly reliable. Among all countries, the CRO - reported PLATO-USA outcomes represent the largest and most realistic dataset of realistic evidence suggesting ticagrelor inferiority to clopidogrel for all primary endpoint components including vascular death.

## INTRODUCTION

The *PLATelet Inhibition and Clinical Outcomes *(PLATO) trial enrolled 18,624 patients between 2007 and 2009 with moderate to high-risk acute coronary syndromes undergoing coronary intervention or medically managed and were randomized to ticagrelor 180 mg loading dose followed by 90 mg twice daily, or clopidogrel 300- 600 mg loading dose followed by 75 mg once daily, for up to 12 months. The primary endpoint was the time of the first event of death from vascular causes including fatal bleeding, myocardial infarction (MI), or stroke, and occurred in 11.7% of patients treated with clopidogrel, versus 9.8% of patients randomized to ticagrelor (HR=0.84; CI=0.77-0.92; p<0.001)^1^. Remarkably, ticagrelor, being a novel oral, reversible, direct-acting inhibitor of the adenosine diphosphate receptor P2Y12 reduced death from vascular causes (4.0% vs. 5.1%, P = 0.001), and death from any cause (4.5%, vs. 5.9% P<0.001)^2^. Such “mortality wonder” was unexpected and has been challenged by neutral Phase II DISPERSE study results, mismatch between PLATO myocardial infarction and death rates, inverted US outcomes, paradoxical delayed timing of benefit, last minute change of trial monitoring, introduction of electronic clinical research forms (eCRF)^2^, and few striking errors picked up and reported by the FDA reviewers^3^. However, no hard evidence with the reliable proof of data misreporting was available in public domain for over a decade. As of today, ticagrelor holds a superiority recommendation over clopidogrel for acute coronary syndromes in European^4^, Canadian^5^ and American^6^ guidelines based predominantly on the PLATO trial results^1^. We recently gained access to the detailed dataset of 938 PLATO deaths reported to the FDA and matched those records with local patient-level data for 53 deaths from 15 sites in 7 countries controlled by the sponsors. We found that actual existence, precise dates and proper causes of some deaths in PLATO were inaccurately reported^7^. Several clopidogrel deaths were reported earlier than actual, while their causes were switched from “non- vascular” or “unknown” to “vascular”. In contrast, few ticagrelor deaths were omitted or reported later while some vascular deaths were incorrectly entered into the FDA list as “non-vascular” or “unknown”^7^. Realizing that even PLATO Investigators acknowledged that the advantages of ticagrelor over clopidogrel were inconsistent and exhibited “geographical” differences^8^, such discrepancy may be heavily depending upon whether or not the sponsors had been involved in site monitoring in certain countries^2,9^. We here compare if there were any differences when PLATO deaths were reported by the sponsors versus independent CRO.

## METHODS

Based on the Freedom of Information Act, we filed a legal complaint in a US federal court, won an expedited order, and obtained the complete PLATO death list submitted to the FDA by the ticagrelor NDA 22-433 sponsor. The FDA spreadsheet contains 938 PLATO deaths with trial ID numbers, country, enrolling site, patient age, gender, treatment assignments, discontinuations, outcome codes, dates and precise causes of trial exit. Each event contains whether the death cause was vascular (code 11), non- vascular (code 12), or unknown (code 97). There were 14 subcodes for vascular, 9 subcodes for non-vascular deaths, and universal code “99” which applied for “other” causes. We triaged all PLATO reported deaths into vascular and non-vascular cohorts and applied above-mentioned codes to each event. Most of the data were controlled and reported by PLATO sponsor, with the exception of the USA, Russia, Georgia, and most (sites 5101-5106) of Ukraine. The entire US was monitored by ReSearch Pharmaceutical Services, (Wort Washington, Pennsylvania, USA; http://www.rpsweb.com). All Russian, Georgian, and most Ukrainian sites were monitored by Evidence CRP, now Worldwide Clinical Trials, (Morrisville, North Carolina, USA; http://wwctrials.com/). The combined CRO data were matched with the rest of PLATO (39 countries and site 6301 from Ukraine) which were sponsor monitored.

## RESULTS

**Sponsors versus CRO reporting:**

**Figure 1:**
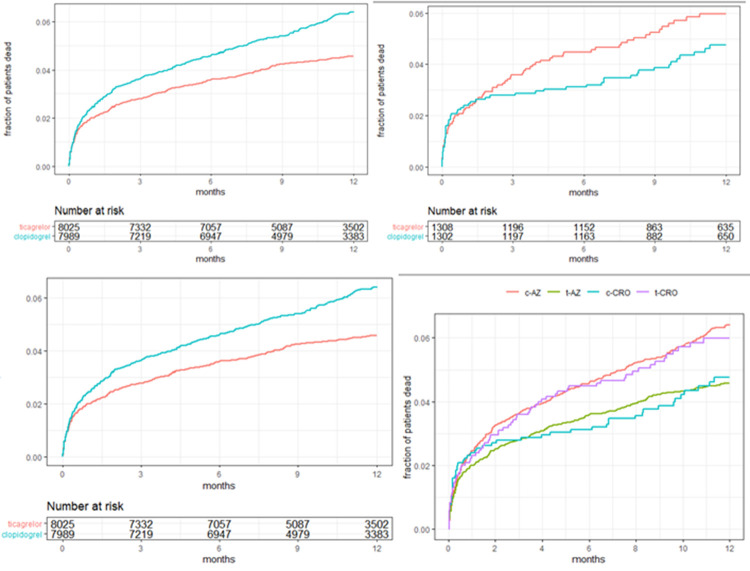
Sponsors versus CRO death reporting in PLATO Kaplan-Meyer mortality curves over 1-year follow-up reported by sponsor (**A**), CRO (**B**), total (**C**), and exposure of A over B (**D**) suggesting inverted death risks dependent on monitoring source.

Among 938 PLATO-FDA deaths sponsors reported 807, and CRO reported 131 exits; overall 684 of them were vascular, 161 non-vascular, and many (n=93) of unknown origin. With regard to the source-reporting differences CRO reported slightly more vascular deaths than the sponsors, although their numbers were small. The rate of “unknown” deaths was high but similar between sources. The details are outlined in Table 1. Overall, the sponsor versus CRO death reporting to the FDA in PLATO are now confirmed in the Kaplan-Meyer mortality curves over 1-year follow-up suggesting inverted death risks dependent on monitoring source (Figure 1).

**Table 1:** Deaths reported to FDA in PLATO by cause and monitoring source CRO – Contract Research Organization; C/T – clopidogrel/ticagrelor

Trial Monitoring	Vascular (code “11”) %, C/T	Non-Vascular (code “12”) %, C/T	Unknown (code “97”) %, C/T
CRO	102; 77% (45/57)	15; 12% (7/8)	14; 11% (4/10)
Sponsor	582; 72% (328/254)	146; 18% (87/59)	79; 10% (53/26)
Total	684 (373/311)	161 (94/67)	93 (57/36)

**Differences in the death cause: **With regard to vascular deaths reporting the significant differences were observed between sources. PLATO sponsors reported more sudden (71 vs. 52; p<0.001), heart failure (56 vs.40; p<0.007) and arrhythmia deaths (25 vs.18; p=0.04) after clopidogrel. None of these trends were observed in the CRO reporting, moreover, there were more post-MI deaths (27 vs.15; p=0.01) after ticagrelor. Importantly, ticagrelor was numerically inferior for 6 vascular subcodes, and superior only in non-trauma bleeding. In contrast, similar comparison among the sponsors-reported vascular deaths suggests that ticagrelor was better for 6 subcodes. The details of vascular deaths distributions dependent on reported sources are presented in Table 2.The non-vascular death reporting was also quite different in PLATO heavily dependent on the source. The details are outlined in Table 3. Among 9 subcodes reported by the sponsor the most notable were more cancer (17 vs. 11; p=0.02); sepsis (23 vs.7; p=0.003); and multiorgan failures (13 vs. 7; p=0.001) as deaths causes against clopidogrel. These significant differences were lacking in the CRO reported pool although the numbers of events are small.

**Table 2:** Vascular deaths reported by CRO and Sponsor in PLATO CAD – coronary artery disease

Cause	PLATO Code	Clopidogrel CRO (n=45)	Ticagrelor CRO (n=57)	Clopidogrel Sponsor (n=328)	Ticagrelor Sponsor (n=254)
Sudden Death	11-1	7	8	71	52
Myocardial Infarction	11-2	15	27	75	61
Unstable angina	11-3	-	3	10	5
Other CAD	11-4	-	-	4	4
Stroke	11-5	1	2	17	19
Arterial Embolism	11-6	1	1	1	-
Pulmonary embolism	11-7	1	-	7	2
Ruptured aortic aneurism	11-8	-	1	-	-
Aortic dissection	11-9	-	-	-	-
Heart failure	11-10	6	7	56	40
Cardiac arrhythmia	11-11	3	5	25	18
Bleeding (not trauma)	11-12	2	-	13	12
Endocarditis	11-13	-	-	-	1
Valvular disease	11-14	-	-	1	-
Other	11-99	9	3	48	40

**Table 3:** Nonvascular deaths reported by CRO and Sponsor in PLATO

Cause	PLATO Code	Clopidogrel CRO (n=7)	Ticagrelor CRO (n=8)	Clopidogrel Sponsor(n=87)	Ticagrelor Sponsor (n=59)
Respiratory failure	12-1	3	1	9	12
Pneumonia	12-2	-	1	17	9
Cancer	12-3	-	3	17	11
Trauma	12-4	1	-	-	3
Suicide	12-5	-	-	1	1
Liver failure	12-6	-	-	1	-
Renal failure	12-7	-	-	5	2
Sepsis	12-8	1	-	22	7
Multiorgan failure	12-9	1	2	13	7
Other	12-99	1	1	10	7

**Figure 2:**
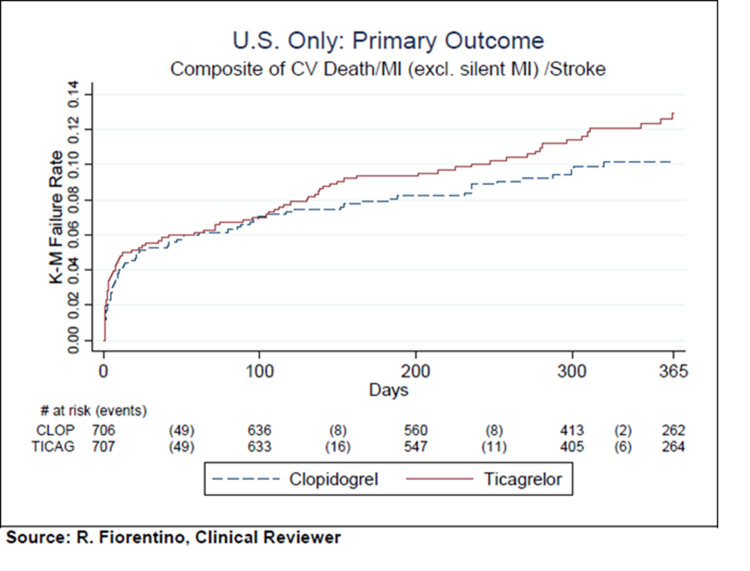
FDA generated primary endpoint in PLATO-USA

**PLATO-USA deaths: **Summarizing all available data, it seems the United States outcomes among 1,413 patients currently represent the largest and most valuable dataset of sponsor-free pooled data suggesting ticagrelor inferiority to clopidogrel for all PLATO primary endpoint components^3^. The FDA efficacy reviewer for ticagrelor NDA 22-433 counted primary endpoint events in PLATO-USA, and these data are presented in Figure 2.

## DISCUSSION

The main findings of this analysis are that PLATO sponsors and CRO reported substantially different and inverted patterns of mortality trends after ticagrelor or clopidogrel within the same trial. Among the reported causes of deaths most differences were observed for sudden myocardial infarction, heart failure, sepsis, cancer and multiorgan failure requiring further exploration for matching of the outcomes reported to the FDA versus confirmed local patient-level evidence. Importantly, all mortality advantages of ticagrelor were reported exclusively by the sponsors-controlled sites and countries. In contrast, the CRO-monitored sites reported more ticagrelor deaths than after clopidogrel in US (29/24), Russia (29/19) and Georgia (12/7), but not in Ukraine (5/6) for the total of (75 vs. 56, p<0.01). The CRO revealed no ticagrelor mortality benefit for any clinical cohort dissected by 14 vascular and 9 non-vascular PLATO death codes. Since we now know that details of some deaths such as causes, precise timing and actual event occurrences were inaccurately reported by the sponsors in PLATO favoring ticagrelor^7^ it was important to outline differences (if any) in deaths reporting between the sponsors and independent CRO which were in charge of about 15% of enrolled patients. Should the CRO death reporting be in agreement with the sponsor that would raise assurance that the problems we observed from the relatively small subset of 53 deaths^7^ were minor and would not impact the results of the entire PLATO trial. Unfortunately, the differences between reporting sources are striking, and clearly indicative that declared and currently accepted ticagrelor benefits in PLATO are exaggerated and derived exclusively from the sponsor-controlled sites. Noticeable, some vascular and non-vascular deaths codes applied in PLATO were questionable, and their structure should be probably redesigned for future trials. Indeed, 93 deaths of “unknown” (code 97) appears excessive as well as additional 100 vascular deaths of unclear cause (code 11-99) was not a success. Additionally, 19 unclear non-vascular deaths (code 12-99) makes the list of subcodes pretty useless when over 200 out of 938 deaths in the indication-seeking trial cannot be precisely identified and properly recorded. We paid special attention to the sudden death since it was the most frequent outcome in our comparison between local patient-level site evidence and data submitted to the FDA^7^. Indeed, among 53 deaths, 4 clopidogrel patients were upgraded to, and 4 ticagrelor patients escaped proper sudden death reporting^7^. That mismatch occurred in all countries where we have an ability to retrieve local data. Analyzing sudden death reporting revealed that CRO numbers are unremarkable, but sponsor yielded significant benefit of ticagrelor. The reasoning behind such obvious trends is not exactly clear. However, it may be attributed to the inclusion of extra vascular deaths to the PLATO primary endpoint. Another disparity is much less post-AMI deaths reported by the sponsor than by CRO. What we know now is that some sudden deaths (code 11-1) were in fact post-AMI (code 11-2), but the value of 11-2 code was less desirable since it would be counted as AMI for the primary efficacy endpoint limiting ticagrelor “death prevention” benefit Since AMI rates in PLATO were reasonable^1^, and not inflated as deaths, it could suggest that the sponsor was working in tandem with the International Central Adjudication Committee. Indeed, centrally-adjudicated AMI numbers showed a remarkable discrepancy to site- reported AMI’s by more than a doubling of the difference: from 44 to 89 events in favor of ticagrelor in PLATO (from a HR=0.94, p=0.095; to a HR=0.84, p<0.001)^10,11^. So, in lay terms, sudden death as an outcome was most precious for targeting mortality benefit and was inflated against clopidogrel in PLATO to the full extend. Regarding heart failure deaths reporting mismatch, it is unclear how this code could have been manipulated. Few PLATO patients who died at nursing homes, or at advanced age had multiple comorbidities, often including heart failure. Those who died at home were adjudicated dependent on their treatment arm. Clopidogrel patient’s cause of death was reported as heart failure (code 11-10), but if the patient received ticagrelor non-vascular co- morbidity was chosen as a primary death cause. Therefore, deceased patients with very similar array of diseases may have been reported as vascular or non-vascular primary death cause. Another important issue is lack of the potential consistency of ticagrelor mortality benefit reported in PLATO. The indirect evidence suggests quite the opposite. No other prospective large study reported ticagrelor deaths reduction in various settings and populations cohorts. Moreover, the industry- sponsored ATLANTIC trial revealed that prehospital administration of ticagrelor in patients with acute STEMI appeared to be safe but did not improve pre- PCI coronary reperfusion^12^. The ATLANTIC data fully support the PLATO Angiographic substudy^13^ denying early benefit of ticagrelor and correspond well with lack of immediate clinical benefit including the early PCI "death paradox" in PLATO-USA patients. Finally, there were significantly (p=0.043) more deaths in early ticagrelor ATLANTIC arm (odds ratio 3.18 (1.02- 9.90) challenging stent thrombosis reduction). In contrast to PLATO, in the PEGASUS there was identical all-cause mortality (RR=1.00; 95%CI 0.86- 1.16, p=0.99) versus placebo and unexplained late addition of 198 primary events suggestive of data manipulation^14^. Finally, the East-Asian PHILO trial revealed numerical inferiority of ticagrelor with regard to death, myocardial infarction, stroke, and bleeding over clopidogrel very similar to the PLATO-US evidence^15^. Finally, declared ticagrelor benefits for reduction of cancer^16^ and sepsis^17^ deaths were severely challenged since they were reported in multiple pairs, or mismatched with local evidence. There are obvious limitations to our report worth mentioning. We were able to retrieve and verify only few data from only 861 patients, with 53 deaths. These 53 deaths were verified with sites and their PI’s regarding causes and exact dates. Most PLATO investigators, however, refused to verify outcomes being under heavy industry pressure. Thus, most PLATO fatalities are still unverified and current numbers may not be high enough to be absolutely compelling to precisely assess the magnitude of the problem. However, the differences in CRO versus sponsors reporting are striking. It is quite possible that some site records were inaccurate, but we tried to use at least two sources to verify local evidence. The differences in record keeping quality heavily depends on participating countries, with the top scores belonging to Mexico and Canada. Importantly, beyond PLATO there are numerous large datasets suggesting no ticagrelor mortality benefit over clopidogrel, especially in the elderly and East Asian cohorts. With regard to study limitations the data monitoring in pharma-industry led trials should be observed by CROs rather the sponsors, the relevance of the data presented may be rather limited. Several similar manuscripts focusing on reported/ misreported outcome data in the PLATO trial have already been published. However, we never analyzed the evidence as sponsor versus CRO outcomes.

## CONCLUSION

We conclude that deaths were reported differently by sponsors and CRO within the same trial. Since some deaths were misreported by PLATO sponsors, only the CRO data seems mostly reliable. Among all countries, the CRO - reported PLATO-USA outcomes represent the largest and most realistic dataset of realistic evidence suggesting ticagrelor inferiority to clopidogrel for all primary endpoint components.
